# Clinical Application of Human Mesenchymal Stromal Cells for Bone Tissue Engineering

**DOI:** 10.4061/2010/215625

**Published:** 2010-11-11

**Authors:** Anindita Chatterjea, Gert Meijer, Clemens van Blitterswijk, Jan de Boer

**Affiliations:** ^1^Department of Tissue Regeneration, MIRA Institute for Biomedical Technology and Technical Medicine, University of Twente, 7500 Enschede, The Netherlands; ^2^Department of Periodontology and Biomaterials, Radboud University Nijmegen Medical Center, 6525 Nijmegen, The Netherlands; ^3^Department of Oral and Maxillofacial Surgery, Radboud University Nijmegen Medical Center, 6500 Nijmegen, The Netherlands

## Abstract

The gold standard in the repair of bony defects is autologous bone grafting, even though it has drawbacks in terms of availability and morbidity at the harvesting site. Bone-tissue engineering, in which osteogenic cells and scaffolds are combined, is considered as a potential bone graft substitute strategy. Proof-of-principle for bone tissue engineering using mesenchymal stromal cells (MSCs) has been demonstrated in various animal models. In addition, 7 human clinical studies have so far been conducted. Because the experimental design and evaluation parameters of the studies are rather heterogeneous, it is difficult to draw conclusive evidence on the performance of one approach over the other. However, it seems that bone apposition by the grafted MSCs in these studies is observed but not sufficient to bridge large bone defects. In this paper, we discuss the published human clinical studies performed so far for bone-tissue regeneration, using culture-expanded, nongenetically modified MSCs from various sources and extract from it points of consideration for future clinical studies.

## 1. Introduction

**Figure 1:**
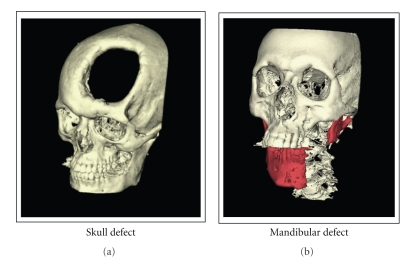
3D reconstruction of a skull and mandibular defect in trauma patients. Surgeons are often faced with patients having large defects in the bone which do not heal spontaneously. The gaping hole in the skull and the area highlighted in red in the mandible are examples of large-sized defects in real patients. Though autografts are the gold standard treatment for such patients, the amount of graft material required is often the limiting factor. Tissue regeneration using synthetic or natural scaffolds seeded with mesenchymal stem cells can be an alternative solution for such patients.

**Figure 2:**
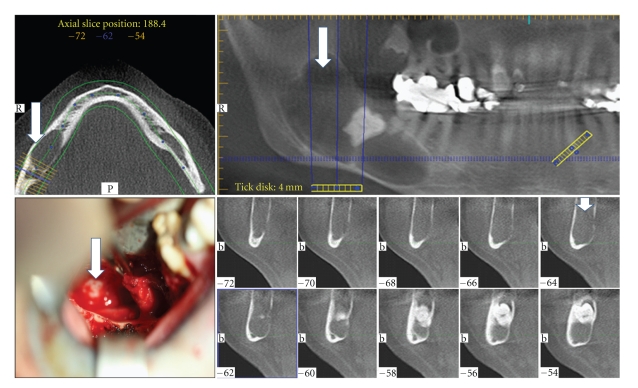
Mandibular defect following cyst. CT scan of huge cyst in the mandible (see white arrows). The clinical picture represents the situation after removing the cyst revealing the alveolar nerve positioned at the bottom of the cavity (black arrow).

Bone lesions/defects caused by, for example, trauma, bone resection due to ablative surgery, or correction of congenital deformities are a common problem in clinical practice. In the majority of the cases, the body's self-healing capacity is able to repair the defect. Yet every year, in roughly 1 million cases of skeletal injury, the defect size is too big or conditions not optimal to allow healing (Figures 1 and 2). In these cases, external help is required in the form of bone graft procedures to achieve union [1]. 

The most frequently used sources of bone grafting are autologous and allogeneic bone [2]. Autologous cancellous bone grafts are most successful in the present clinical scenario, because in addition to being osteoconductive and osteoinductive, they are safe, cheap, and easily available to the surgeons. However, obtaining autologous grafts requires the patient to be subjected to additional surgery, thus introducing extra morbidity at the donor site and increasing surgical costs [3–5]. Besides, the amount of graft material is limited and chances of complications such as infections, instability, and paraesthesia at the donor site can affect up to 30% of patients [6–8]. An alternative is allogeneic bone grafting, which can be obtained from authorized tissue banks which collect and store bone tissues from human cadavers [7]. By this approach, problems associated with harvesting and quantity of graft material are bypassed. To avoid problems associated with immunogenicity, donor grafts can be devitalized via processes such as irradiation and freeze drying. Unfortunately, this processing also eliminates the cellular component, thus reducing the graft's osteoinductivity, thereby resulting in a slower rate of new bone formation as compared to autologous grafts [9].

As an alternative to autologous or allogeneic bone grafts, surgeons may use scaffolds made of synthetic or natural biomaterials that promote the migration, proliferation, and differentiation of bone cells. In the last decade, a large number of publications have illustrated the osteoinductive and osteoconductive properties of scaffolds such as synthetic hydroxyapatite (HA) [10–12], coralline HA [13–15], *β*-Tricalcium phosphate and porous biphasic calcium phosphate [16–19], calcium phosphate cements [20], chemically treated titanium [21], and glass ceramics [22]. However, the degree of osteogenic and osteoinductive properties provided by the osteoprogenitor cells, as present in the autografts, exceeds that of the scaffolds. To improve osteoinductivity, scaffold materials can be loaded with osteoinductive growth factors such as bone morphogenetic proteins (BMPs). The drawback of the growth factor approach are the supraphysiological concentrations needed to obtain the desired osteoinductive effects, their high costs, and more importantly, potential ectopic bone formation [23, 24]. 

Alternatively, scaffolds can be loaded with osteoprogenitor cells in order to generate a living bone graft *in vitro*, an approach referred to as bone-tissue engineering. Various possible sources for osteoprogenitor cells have been considered. Osteoblasts obtained from autologous bone biopsies and then expanded *in vitro* were an obvious first choice due to their nonimmunogenicity. However, the relatively low number of cells obtained after dissociation of the biopsy specimen, the time-consuming nature of the whole process and the problems associated with obtaining osteoblasts from patients with bone-related diseases prompted continuation of the search for better options [1, 25]. Mesenchymal stromal or stem cells (MSCs) which can be obtained from various tissue sources, like bone marrow, adipose tissue, umbilical cord, or placenta provide an alternative source of osteoprogenitor cells. MSCs were first identified in the bone marrow by Friedenstein and coworkers in 1966 [26] and were subsequently named mesenchymal stem cell (MSCs) by Caplan [27]. They are very attractive to researchers as they can be extensively expanded *in vitro* to obtain numbers sufficient to treat large bone defects [28], and they have immunosuppressive effects *in vivo,* which may make them suitable for allogeneic transplantations [29, 30]. MSCs isolated from different sources share many phenotypical and functional characteristics. However, depending on the tissue source and the isolation methods employed, their differentiation potential varies [31]. The varied tissue sources and isolation methods make it difficult to determine if the resulting cells are sufficiently similar to allow for a direct comparison. Therefore, the International Society for Cellular Therapy proposed a set of minimal criteria to label a cell as a MSC [32]. These include: (1) cells must be plastic adherent when maintained in standard culture conditions; (2) they must express CD105, CD73, and CD90 and lack expression of CD 45, CD34, CD14, or CD11b, CD79*α* or CD 19 and HLA-DR surface molecules; (3) they should differentiate into osteoblasts, adipocytes, and chondroblasts *in vitro*. Haynesworth et al. were the first to combine human MSCs from adult bone marrow with ceramic scaffolds to successfully generate bone *in vivo* upon ectopic implantation into immunodeficient mice [33]. This provided proof-of-principle on the feasibility of using hMSCs in bone-tissue engineering. Since then a lot of interest has been generated in the field of tissue engineering, resulting in *in vitro* and *in vivo* studies with different scaffold/cell combinations. The proof of concept for repair of critically sized bone defects using tissue-engineered bone graft substitutes has been provided by a number of animal studies [30, 34–44], and several clinical studies have been conducted to assess the safety and efficacy of this approach in man. Nevertheless, bone-tissue engineering did not find its way to routine clinical practice. 

Here, we present an overview of all published human clinical studies performed so far to generate bone using constructs seeded with culture expanded, autologous, nongenetically modified MSCs obtained from various human cell sources, suggest possible recommendations for future design of clinical studies, and describe future research directions. Cells from the periosteum have not been included in this paper because no studies have been performed to determine the MSC nature of the periosteal cells used in the clinical studies. There are previous reports which indicate that the periosteal cells fulfill the minimal criteria to be labeled as an MSC [45–48]. However, there are differences in the isolation and expansion protocols used in these studies and in the studies employing the cells for clinical applications [49–52]. Studies using the mononuclear fraction of the bone marrow or adipose tissue have also been excluded from this paper. Although MSCs are present in the mononuclear fraction, other populations of cells also form a large part of this fraction.

## 2. Clinical Studies in Humans Using Autologous MSCs from Various Cell Sources for Bone Tissue Engineering

Prior to market release of tissue-engineered products, an investigational new drug application (NDA) may have to be submitted to accredited regulatory bodies such as the Food and Drug Administration (FDA) or the European Medicines Agency (EMEA). Following this, clinical trials have to be enrolled as phase 1, phase 2, or phase 3 trials. In phase 1, evidence is obtained about the safety of a particular approach in a selected group of patients. Generally, these are small trials with a number of patients recruited being less than 30. In phase 2, more patients are included to evaluate effectiveness on the possible applications. Phase 3 clinical trials involve multicentre trials on 300–3000 patients and are a definitive assessment of the concerned treatment in comparison with the current gold standard. Following completion of all phases of clinical trials, the regulatory body reviews the results, before making a final decision on the release of the tissue engineered product in the market. A search on *clinicaltrials.gov *using search terms, “Mesenchymal stromal cells”, “autologous MSCs,” and “bone tissue engineering”, provided 2 relevant studies.

1. “Treatment of osteonecrosis of the femoral head with implantation of autologous bone marrow cells, a pilot study.” This is a phase 1 study, which started in January 1999 and was completed in September 2008. However, no publications describing the study results are currently available in literature.
2. “Autologous implantation of Mesenchymal stem cells for the treatment of distal tibial fractures”. This is an ongoing, phase 1/phase 2 study. The study started in April 2009 and the expected primary completion date is April 2011. No results from this study have yet been published in the literature

Designs of clinical trials vary from randomized control trials (RCT), replicated single subject experiments, cohort outcome studies, systematic case studies, and case reports. In general, the more rigorous the design of a clinical trial, the greater the credibility that can be attached to the conclusions derived from the outcome of a study. Based on the methodological rigour applied, RCTs are generally considered at the top of the hierarchy as randomization in selection of patients for inclusion in the various treatment groups ensures negation of the selection bias while inclusion of controls help rule out the effects of the confounding factors that may have an effect on the treatment outcome. However, due to practical and ethical issues involved in conducting RCTs, most of the trials conducted on human patients and described in literature for bone-tissue engineering are at the level of cohort outcome studies or case reports. Cohort studies provide information on the percentage of patients which respond positively to a given therapeutic technique while case reports describe the effects of using a particular tissue-engineered graft in a single patient. The observed results in the latter can be thus idiosyncratic to the specific patient being evaluated and systematic replications of the experiment would be necessary prior to obtaining conclusive evidence. Absence of controls in cohort studies is a major drawback of such a study design. However, these preliminary attempts also have an important role in the development of scientific research because they generate information that can provide some clues to the safety and potential therapeutic effects of the treatment option and may stimulate researchers to perform the more elaborate, time consuming, and costly RCTs in the future. In this paper, we list all human clinical studies, including case reports that have been published in literature, using autologous, culture expanded, nongenetically modified, human MSCs for purpose of bone tissue engineering. None of these studies have obtained approval from institutions such as FDA or EMEA. The ethical approval for conducting these studies has been provided by their respective local university/hospital ethics committees. 

**Figure 3:**
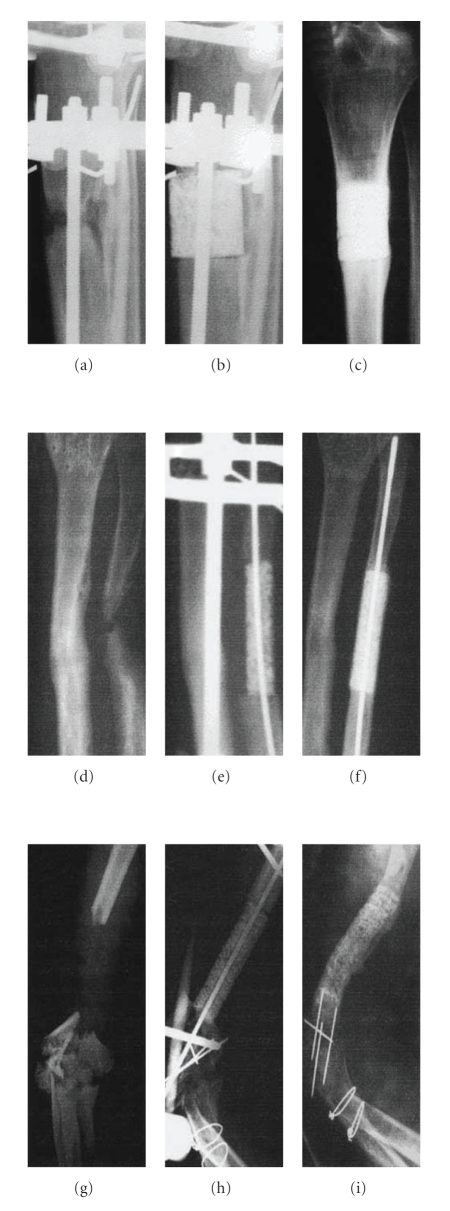
Radiographs obtained before and after the repair of large bone defects in three Patients from the study by Quarto et al. panels (a)–(c) show films obtained from Patient 1 before, immediately after, and 18 months after surgery, respectively. Panels (d)–(f) show films from Patient 2 before, immediately after, and eight months after surgery, respectively. Panels (g)–(i) show films from Patient 3 before, immediately after, and 15 months after surgery, respectively. All the films obtained at the last time point demonstrate bridging of the defect with newly formed bone.

The first clinical case series demonstrating feasibility of using tissue-engineered constructs (TEC), as an alternative to autologous bone grafts for treating long bone defects, was reported by Quarto et al. [53]. In 2001, they described the preliminary results of 3 patients (27, 16, and 15 months respectively postsurgery) suffering from various segmental defects (Figure 3). The patients were chosen because conventional surgical therapies such as Ilizarov's technique which excludes graft transplant, had failed. The Ilizarov's technique relies on the bone regeneration potential to fill the gap created artificially via osteotomy of the affected segment while maintaining the periosteum intact and then distraction of the two separated halves fixed apart used ring fixators [54]. Autografts were technically difficult to perform because the degree of bone loss would leave the patient with serious donor site morbidity. The first patient was a 41-year-old female with a 4 cm large segmental bone defect in the right tibia, the second a 16-year-old female suffering from a traumatic loss of a 4 cm segment of the distal diaphysis of the right ulna, while patient 3 was a 22-year-old male, who missed a 7 cm segment of the right humerus. For all the patients, macroporous 100% hydroxyapatite (HA) scaffolds were custom made to fit the shape and size of the defect. These were then loaded with *ex vivo* expanded hMSCs isolated from their own bone marrow. All 3 patients were monitored with radiographs and CT scans, which revealed abundant callus formation by the second month postsurgery and good integration of the implants with peri-implant bone formation by the sixth month after surgery. A followup report 6-7 years after surgery reported that the implants displayed good osseointegration with no further complications. Angiographic evaluation performed in patient 3, 6.5 years after surgery also indicated vascularization of the grafted zone suggesting presence of vital bone at the graft site. However, no controls were included in this study, and initial followup was based only on radiological evaluation which the authors admit was not optimal because the high mineral density of the scaffolds used made it difficult to differentiate the new bone from the preexisting scaffold [55]. Nevertheless, the study showed that the procedure is safe to perform.

In the years after the initial trial, case studies involving single patients treated with tissue-engineered constructs were reported in the literature. In 2007, Krecic Stres et al. treated 1 patient with a comminuted fracture femur using a combination of TEC and autologous cancellous bone in a ratio of 2 : 1 [56]. The TEC was generated by seeding bone marrow derived MSCs on porous calcium-triphosphate granules. Clinically, the researchers claim that the patient has been recovering well. However, the combination of autologous bone with the TEC makes it difficult to draw conclusive inferences on the feasibility of using TEC alone for bone-tissue engineering as it would be impossible to determine the individual contributions of the TEC and the autologous bone. Moreover, the investigators only relied on clinical evaluation and X-rays to determine new bone formation. No controls or biopsies were planned for the patient. Also, the actual defect size was not mentioned. This is essential as the size of the graft has been found to be crucial in determining the survival of the cells within the core of the graft.

Hibi et al. reconstructed an alveolar cleft defect by injecting culture expanded and osteogenically-induced bone marrow derived MSC mixed with autologous platelet rich plasma [57]. This study provided a novel approach of using autologous platelet rich plasma as the scaffolding material for the cells. The patient was followed up postoperatively with serial CT scans which showed the regenerated bone extending from the cleft walls after 3 months and bridging the cleft after 6 months. It remains unclear whether the defect is filled by bone tissues produced by the implanted cells, or it is formed due to osteoconduction from the border of the cleft defect.

In 2010, Lee et al. described a successful reconstruction followed by dental implant placement of a 15 cm jaw defect as a result of segmental mandibulectomy due to central hemangioma in a 14-year-old boy [58]. Three reconstructive surgeries were performed. In the first surgery, autologous resected mandible obtained during the hemimandibulectomy was used as a tray into which osteogenic-differentiated autologous bone marrow stem cells and fibrin glue was injected. Due to lack of adequate mandibular bone for dental implant placement and recovery of dentition, the second surgery involved vertical distraction osteogenesis with injection of autologous osteoblastic-differentiated MSCs. The third and final surgery was 7 months later for implant placement. At the time of implant placement biopsies were taken from the implant site and histological evaluation of the biopsies revealed newly formed viable lamellar bone. Dental CT images taken 4 months after the implant placement confirmed continued presence of mineralized bone at the augmentation zone. 

**Figure 4:**
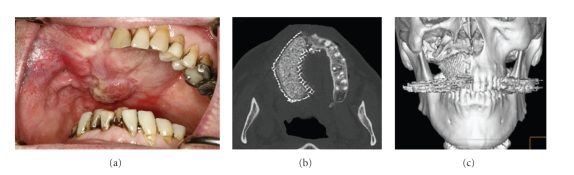
Two months postoperative results of the study by Mesimaki et al. reconstructed a major maxillary defect in an adult patient using autologous adipose-derived MSCs (ASCs) combined with rhBMP-2 and *β*-TCP granules in a microvascular reconstruction surgery. Two months postoperative results indicate that (a) the rectus abdominis muscle has atrophied nearly totally and epithelialized almost completely. Only a small area in the molar region was nonepithelialized. A well-formed buccal sulcus is also noted. Axial (b) and 3D CT scans (c) show the shape and normal bone density of the new maxilla.

In 2009, Mesimäki et al. reconstructed a major maxillary defect in an adult patient using autologous adipose-derived MSCs (ASCs) combined with rhBMP-2 and *β*-TCP granules in a microvascular reconstruction surgery [59]. After isolating the ASCs from abdominal subcutaneous fat in autologous serum using GMP class clean room facilities, the cells were seeded on *β*-TCP scaffolds. Prior to combining with cells, the scaffolds were incubated for 48 hours in basal medium supplemented with rhBMP-2. This medium was discarded when the cells were added and fresh medium without rhBMP-2 was added. The cell scaffold combination was kept in culture for 48 hours prior to their placement in a titanium cage and subsequent implantation in a pouch prepared in the patients left rectus abdominus muscle. The vascular supply of the muscle was not disturbed. 8 months later, the rectus abdominus muscle pouch was opened, and the titanium cage filled with the TCP granules and ASCs was macroscopically examined. The new bone formed in the cage was clinically confirmed to be vital and rigid. A biopsy taken from the newly formed bone revealed histology of normal mature bone. Subsequently, the vessels were disconnected from the rectus abdominus muscle and the muscle flap together with the tissue engineered bone was placed in the maxillary defect. The abdominal vessels were reanastamosed with the facial vessels. The muscle was left to epithelialize intraorally. The patient was followed up with CT scans. Within two months of the surgery, the muscle flap had almost completely epithelialized, and the shape and normal bone density was achieved in the reconstructed maxilla (Figure 4). Four months after placement of the graft, dental implants were placed and their primary stability was reported to be excellent. The implants osseointegrated without any reported adverse effects. This study was the first clinical case where ectopic bone was produced using autologous ASCs in a microvascular reconstruction study. It demonstrated the feasibility and safety of using ASCs for bone regeneration. However, the relative contribution of the rhBMP-2 and the ASCs in the new bone formed remains to be determined. 

In 2007, a study was performed by Sbayesteh et al. for posterior maxillary sinus augmentation involving 6 patients [60]. In this study, the cell source was the bone marrow from the iliac crest and the carrier material was hydroxyl apatite/*β*-tricalcium phosphate (HA/TCP) particle. After 3 months, biopsies were taken and results showed a mean bone formation of 41%. Although biopsies were taken, no information of the bone distribution in the scaffold or the source of the newly formed bone (donor or recipient) was provided. 

**Figure 5:**
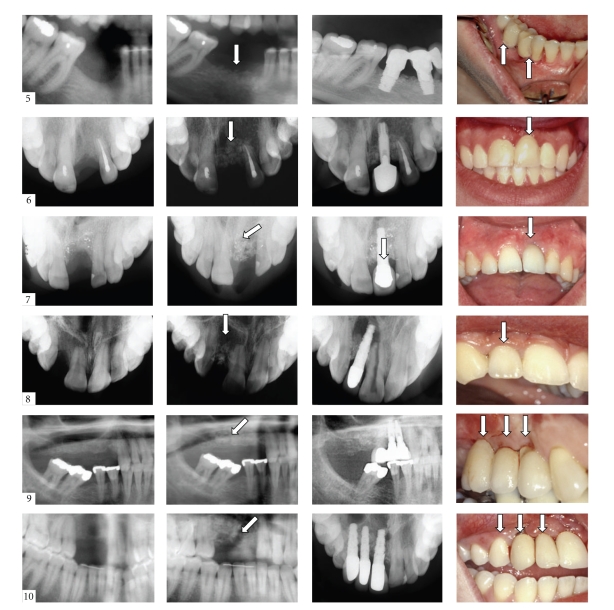
Overview for patients 5–10 from the study by Meijer et al. to reconstruct a maxillary defect and placement of dental implants. First column; radiographs showing the alveolar defects. Second column; showing the reconstruction (arrow) by augmentation (5–8) and by sinus elevation procedure (9 and 10). Third column; radiographs showing the dental implants and the prosthetic construction (crown or bridge). Fourth column; clinical situation at the end of the rehabilitations (arrow).

Another clinical study was reported by our group in order to test the potency of bone-tissue engineering using bone marrow-derived MSCs seeded onto hydroxyapatite particles in 6 patients, requiring reconstruction of bony jaw defects prior to dental implant placement (Figure 5) [61]. Culture expanded bone marrow-derived MSCs were seeded on hydroxyapatite particles varying in size from 1–4 mm^3^. Similar to the work of Schimming et al., the cells were grown on the scaffolds for another 7 days in order to allow further osteogenic differentiation and extracellular matrix deposition and then placed under the mucoperiosteal flap in the defect site. In this study, both the *in vitro* osteogenic capacity and the *in vivo* bone-forming potential of the constructs was assessed using representative samples of cells and constructs, respectively. The *in vitro* potential was tested by performing alkaline phosphatase staining, while the *in vivo* bone-forming capacity of the constructs was confirmed by implanting representative constructs, prepared in an identical fashion to the constructs actually used for the defects, in subcutaneous locations in nude mice. Although no quantification of the bone formed by these hMSCs in the mouse subcutaneous model has been performed, we noted that all the constructs with cells implanted in the nude mice showed bone formation. Four months after application of the construct in the jaw of the human subjects, and before placement of the implant, a biopsy was taken from the operation site. Bone formation was evaluated histologically in the human patients, and in 3 of them, no new bone formation was observed. Of the remaining 3, in 2 patients bone tissue in the scaffolds was observed in close contact with the preexisting bone of the bony defect. This can likely be attributed to migration of osteoblasts from the surrounding bone tissue. In only 1 patient, bone formation was observed more than 7 mm from the preexisting bone tissue. We consider this to be strongly suggestive for *de novo* osteogenesis induced by the implanted cells. An overview of the above mentioned clinical trials are presented in Table 1.

## 3. Experimental Design of Clinical Studies

The clinical studies conducted so far have demonstrated that it is safe to use hMSCs in bone grafting procedures. None of the reports mention adverse effects such as inflammation or excessive tissue growth, despite the fact that there are *in vitro* studies which suggest that MSCs which have been extensively cultured (4-5 months) can develop genomic instability, which can be an indicator of malignant transformation [62–64]. For most clinical applications, a 6–8-week expansion phase provides sufficient cell numbers. This may account for the fact that no malignant potential of the TECs has been observed in the clinical cases performed so far. However, to ensure safety for the patient, we propose that in future clinical studies, chromosomal analysis of implanted cells is assesed. Secondly, most of the clinical studies published have a short followup period. We recommend using longer followup periods to obtain data on the long term safety of TEC.

The data presented in the clinical studies make it likely that the grafted hMSCs were able to contribute to bone regeneration, which provides proof of concept for the potential use of tissue-engineered grafts in bone regeneration. However, the lack of “gold standard” controls and objective evaluation measures such as bone quantification using histology makes it difficult to draw strong conclusions. The studies where biopsies have been used to evaluate the percentage of bone formed seem to suggest that the contribution of the grafted cells is very limited and certainly not sufficient to bridge critical-sized defects. Thus, in order to be able to normalize the efficacy of a given bone TE strategy with respect to that of other trials, we recommend the use of a reference for the bone-forming potential of a tissue-engineered graft. Given that immune-deficient mice have been used by many researchers in the field, we would like to propose that the tissue-engineered grafts to be implanted in patients will be evaluated in mice in parallel and bone formation will be quantitatively assessed. 

Future studies should attempt to include comparisons of the TECs with autologous bone grafts for the same application. This type of study design can thus provide conclusive evidence on the efficacy of the new treatment method as compared to the established standard treatment option. When possible, the two types of implants should be implanted in the same patient. A possible situation when this can be performed without raising ethical issues is when a patient with bilateral defects needs quantities of autologous bone graft which may be difficult to obtain without putting the patient at high risk of complications and morbidity. In such cases, the autograft can be used to treat one defect while the other defect is treated simultaneously with the TEC. 

 Objective evaluation methods should be used to determine the amount of new bone formed. The sample size of the patients should be large enough to allow statistical analysis of the data obtained. We also recommend choice of a surgical site or a tissue-engineered scaffold which allows quantification of bone-tissue formation without added inconvenience to the patient. For instance, we implanted tissue-engineered grafts in the jaw, where we were able to obtain a biopsy in the routine course of the procedure. Other possibilities include tissue-engineered grafts where MRI, microCT or other noninvasive imaging strategies can be applied to quantify bone formation*.*

 In all the clinical studies described, culture-expanded MSCs have been combined with a scaffolding material to generate TECs. Expansion of hMSCs can have unfavorable effects on their differentiation potential [64, 65]. For instance, Banfi et al. demonstrated that as early as after the first passage, the bone forming potential was reduced by about 36 times as compared to fresh marrow [66]. Future studies should employ methods to generate TECs which completely bypass the expansion phase of MSCs on plastic. Studies by Warnke et al. [67], Wongchuensoontorn et al. [68] Gan et al. [69], and Aslan et al. [70] have already demonstrated the feasibility of seeding either mononuclear or enriched populations of MSCs obtained on scaffold material for enhancing the osteogenic potential of the cells. 

## 4. Concluding Remarks

**Figure 6:**
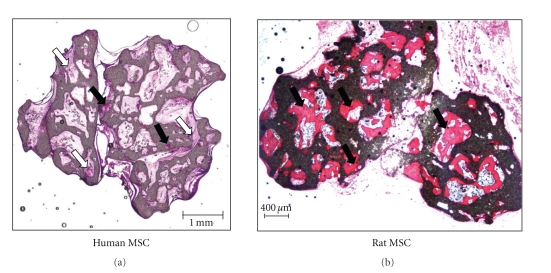
Representative section of scaffold seeded with human bone marrow compared to that seeded with rat bone marrow. Calcium phosphate ceramic scaffolds were seeded with equal number of cells derived from either human or rat bone marrow and implanted subcutaneously in nude mice for 6 weeks. Almost all the pores of the scaffold seeded with rat cells are filled with newly formed bone while the pores of the scaffold seeded with human cells have only one pore with a small amount of bone while the rest of the pores are filled with fibrous tissue. The sections are stained with basic fuschin and methylene blue. The newly formed bone is stained red with basic fuschin (black arrows) while the remaining fibrous tissue is stained pink (white arrows). The black areas represent the scaffold.

Bone-tissue engineering may alleviate problems associated with the current standard treatment used to heal bone defects. However, the success with TECs generated using human MSCs is currently limited. In the majority of the cases, the human MSCs fail to produce clinically relevant amounts of bone while MSCs from other species convincingly generate sufficient bone volume (Figure 6). It is therefore necessary to identify donors with good osteogenic potential and invest research efforts in improving the bone forming capacity of the obtained hMSCs to the level of those obtained from the other species using the widely available ectopic mouse models before embarking on future clinical studies. 

Identification of a donor having cells with good osteogenic potential still poses a major hurdle for bone-tissue engineering. So far, no literature evidence of a positive correlation between hMSC osteogenesis *in vitro* and bone-formation *in vivo* has been reported [71]. Our group isolated hMSCs from 62 donors and assessed the *in vitro* lineage differentiation capacity with gene expression signature and *in vivo* bone forming capacity. We are currently investigating a gene which we believe could be used as a reliable diagnostic marker for *in vivo* bone-forming capacity (unpublished data). 

This is especially attractive as the knowledge that MSCs lack certain surface markers responsible for the host T-cell response opens up possibilities for using such allogeneic cells with proven bone forming potential [72, 73]. In addition to being a ready source of guaranteed bone-forming cells the patient would also have the benefit of not having to undergo immunosuppressive therapy. Moreover, combining allogeneic cells with scaffolds would then make it possible to have a standardized off the shelf bone-tissue engineering product which then can be routinely applied to the clinic. 

Other areas of preclinical research focus should include identification of more potent subfractions of hMSCs, *in vitro* and *in vivo* studies with MSCs isolated from “waste” tissues such as umbilical cord, human placenta, amniotic fluid, and aborted fetuses, alternative seeding strategies to avoid the unphysiological expansion of MSCs on plastic and genetic manipulations of MSCs [74–76] to enhance the expression of osteogenic genes and priming of MSCs using growth factors such as BMPs [77–79] or compounds such as cAMP [43] or vitamin D [80] to enhance the bone-forming capacity while maintaining acceptable costs and safety profile. When the stage is set again for clinical studies, attempts should be made to optimize the experimental design. With the imminent need for bone graft substitutes and the good results obtained with animal-derived MSCs, bone-tissue engineering using human MSCs is likely to reenter the clinic once their biological performance is enhanced.

## Figures and Tables

**Table 1:** Overview of the clinical studies performed on humans using-tissue engineered constructs.

Principal investigator	Year	Cell source	Scaffold	Patients	Area of reconstruction	Salient features	Evaluation	Reported outcome
R.Quarto	2001	Bone marrow	100% hydroxyapatite	3	Long bone defects (1 tibia, 1 ulna, 1 humerus)	(1) First clinical trial in humans using hMSCs (2) Patients with long bone defects selected (3) Patients had good clinical recovery (3) No side effects even after 6-7 years followup	Radiology CT scan Angiography	No quantification of new bone formed. Good integration of the host bone with the implanted scaffolds

H. Hibi	2006	Bone marrow	Platelet gel	1	Alveolar cleft defect	(1) First study using platelet gel as the scaffolding material	Serial Ct scans	Comparable bone formation to that described in literature with autolgous bone grafts

Y.Soleymani	2007	Bone marrow	HA/TCP	6	Maxillary sinus augmentation	(1) Good bone formation in all scaffolds (2) Mean amount of new bone regenerated was 41.3% (3) When compared to the Vacnati study, stark difference in the amount fo bone formed, probably due to location of defect or cell source	Radiology Biopsy	Reported successful with mean bone regenerate as 41.34% and good osseointegration

H.Krecic-Stres	2007	Bone marrow	Porous calcium triphosphate granules	1	Femoral defect	(1) autologous bone graft was mixed with TECs made with MSCs and scaffolds in ratio of 1 : 2 to fill the defect	Radiology	Good clinical recovery. No bone quantification performed

Gert Meijer	2008	Bone marrow	Hydroxyapatite scaffolds	6	Intraoral osseous defects	(1) Only study which performed a biopsy to not just to quantify the amount of bone formed but also the location of bone on the scaffold. This helped identify if the bone was formed due to osteoconduction alone or as a result of osteo conduction with de novo bone sytnthesis. (2) Demonstarted the donor donor variation with hMSCs	Radiology Biopsy	5 patients had no new bone

K. Mesimaki	2009	Adipose tissue	*β*-TCP	1	Maxillary reconstruction	(1) First clinical study to use autologous MSCs derived from adipose tissue and expanded employing good manufacturing protocols (GMP) to heal a bone defect. (2) Use of rhBMP-2 to promote bone formation *in vivo*. (3) Use of a microvascular flap reconstruction surgery for bone tissue engineering	Radiology biopsy	8-month followup indicated presence of mature bone. No quantification of the amount of bone formed is provided. Good clinical course

Jun Lee	2010	Bone marrow	Freeze dried Autologous cancellous bone	1	Mandible reconstruction	(1) Repair of a large segmental defect (15 cm) (2) Injection of MSCs with fibrin glue into the defect site. (3) Use of autologous cancellous freeze dried bone as a tray to hold the MSCs in place.	Biopsy radiology	New bone formation after 4 months. No quantification provided. Goo clinical recovery
